# Microbiomic profiles of bile in patients with benign and malignant pancreaticobiliary disease

**DOI:** 10.1371/journal.pone.0283021

**Published:** 2023-04-18

**Authors:** Shyam K. Poudel, Roshan Padmanabhan, Heloni Dave, Kathryn Guinta, Tyler Stevens, Madhusudhan R. Sanaka, Prabhleen Chahal, Davendra P. S. Sohal, Alok A. Khorana, Charis Eng

**Affiliations:** 1 Department of Hematology and Medical Oncology, Corewell Health William Beaumont University Hospital, Royal Oak, Michigan, United States of America; 2 Department of Medicine, Case Western Reserve University, Cleveland, Ohio, United States of America; 3 Department of Hematology and Medical Oncology, Cleveland Clinic Taussig Cancer Institute, Cleveland, Ohio, United States of America; 4 Department of Gastroenterology, Hepatology and Nutrition, Cleveland Clinic, Cleveland, Ohio, United States of America; 5 Division of Hematology/Oncology, Department of Medicine, University of Cincinnati, Cincinnati, Ohio, United States of America; 6 Department of Genomic Medicine, Cleveland Clinic Lerner Research Institute, Cleveland, Ohio, United States of America; University of Minnesota Twin Cities, UNITED STATES

## Abstract

**Background:**

The prognostic and pathophysiologic significance of the biliary microbiota in pancreaticobiliary malignancies is little understood. Our goal was to find malignancy-related microbiomic fingerprints in bile samples taken from patients with benign and malignant pancreaticobiliary diseases.

**Methods:**

Bile specimens were collected from consenting patients during routine endoscopic retrograde cholangiopancreatography. We used PowerViral RNA/DNA Isolation kit to extract DNA from bile specimens. The Illumina 16S Metagenomic Sequencing Library Preparation guide was used to amplify the bacterial 16S rRNA gene and create libraries. QIIME (Quantitative Insights Into Microbial Ecology), Bioconductor phyloseq, microbiomeSeq, and mixMC packages were used for post-sequencing analysis.

**Results:**

Of 46 enrolled patients, 32 patients had pancreatic cancers, 6 had cholangiocarcinoma and 1 had gallbladder cancer. Rest of the patients had benign diseases including gallstones, and acute and chronic pancreatitis. We used multivariate approach in mixMC to classify Operational Taxonomic Units (OTUs). Doing this, we found a predominance of genera *Dickeya* (p = 0.00008), *[Eubacterium] hallii group* (p = 0.0004), *Bacteroides* (p = 0.0006), *Faecalibacterium* (p = 0.006), *Escherichia-Shigella* (p = 0.008), and *Ruminococcus 1* (p = 0.008) in bile samples from pancreaticobiliary cancers as compared to benign diseases. Additionally, bile samples from patients with pancreatic cancer exhibited a predominance of genus *Rothia* (p = 0.008) as compared to those with cholangiocarcinoma, whereas bile samples from patients with cholangiocarcinoma exhibited a predominance of genera *Akkermansia* (p = 0.031) and *Achromobacter* (p = 0.031) as compared to those with pancreatic cancers.

**Conclusions:**

Both benign and malignant pancreaticobiliary diseases have distinct microbiomic fingerprints. The relative abundance of OTUs in bile samples varies between patients with benign and malignant pancreaticobiliary diseases, as well as between cholangiocarcinoma and pancreatic cancer. Our data suggest that either these OTUs play a role in carcinogenesis or that benign disease-specific microenvironmental changes differ from cancer-specific microenvironmental changes, resulting to a clear separation of OTU clusters. We need more research to confirm and expand on our findings.

## Introduction

Our understanding of the human microbiome in the context of cancer is evolving rapidly. Studies in the past have suggested that close to 16% of global cancer burden could be attributed to infections [1, 2]. Emerging studies reveal the role of the microbiome as a causative, prognostic, and predictive factor in cancer and its treatment [3–5]. For example, *Bacteroides fragilis* and *Escherichia coli* are implicated in colon carcinogenesis in patients with familial adenomatous polyps [6], and the gut microbiome may influence how cancer cells respond to immunotherapy, both for melanoma and epithelial malignancies [7, 8]. However, little is known about the carcinogenic role of bacteria in body sites that are generally considered sterile [9]. Specifically, in pancreaticobiliary cancers, which arise from organs typically not harboring microbes, there is some evidence that the microbiome may play a role in cancer initiation and progression, as well as recovery from surgery and response to therapy [10, 11]. Indeed, recent surveys of human tumor samples suggest both the presence of tumor-residing bacteria in non-mucosal sites including pancreas, breast, ovary, lung and skin; and correlation of microbiome’s metabolic functions to their specific tumor types [12–14]. However, there is limited clinical data on microbiota in pancreaticobiliary malignancies and their most intimate environment—bile.

In patients with benign pancreaticobiliary diseases, a recent study evaluated the microbial communities in bile of patients with primary sclerosing cholangitis (PSC) to suggest the prevalence of *Prevotella*, *Streptococcus*, *Veillonella*, *Fusobacterium*, and *Haemophilus* in the biliary tract and the pathogenic role of *Streptococcus* in disease progression [15]. Both culture-dependent and independent techniques in the past have demonstrated the presence of a microbial community in the human gallbladder and the bile duct in some benign hepatobiliary diseases including acute cholecystitis and gallstones [16–20]. There remains a knowledge gap in our understanding of a “normal” biliary microbiome due to obvious ethical difficulties of obtaining bile from healthy individuals who do not have any hepatobiliary pathologies. A recent study that tried to address this by evaluating bile obtained from liver donors without any record of biliary or hepatic disorders showed abundance of sequences belonging to the family *Propionibacteriaceae* in healthy controls compared to patients with cholelithiasis who had abundance of sequences belong to the families *Bacteroides*, *Prevotellaceae*, and *Veillonellaceae* [21].

Studies from animal models and human beings in the past have suggested that the biliary tract might harbor an indigenous microbiome that might be well adapted to face the local environmental challenges of the biliary microecosystem [21, 22]. However, the question on how this microbiome responds to or contributes to the local inflammatory and neoplastic processes in the pancreaticobiliary tract is not quite fully understood. We conducted an extensive analysis of the bile collected from a series of patients with benign and malignant pancreaticobiliary diseases to identify and correlate the unique signatures of such microbiome to the underlying disease process.

## Materials and methods

We enrolled 46 patients in this study approved by the Cleveland Clinic Institutional Review Board. All patients provided written informed consent. Bile samples were collected from the common bile duct during routine endoscopic retrograde cholangiopancreatography (ERCP).

### DNA extraction

Total DNA was extracted from bile specimens using PowerViral RNA/DNA Isolation kit according to the manufacturer’s protocol (Mo Bio Laboratories, Carlsbad, CA) with minor modifications. Bile specimens were centrifuged at 1500rpm for 10 minutes, and pellets were resuspended in 650 ul MoBio PV1 solution. Samples were then transferred to PowerViral glass bead tubes and warmed at 55° C for 10 minutes. Samples were homogenized using the TissueLyser LT (Qiagen, Valencia, CA) at 25 Hz for 10 minutes and centrifuged at 13,000 x g for 1 minute, after which supernatants were transferred to a clean 2 ml collection tube with 150 uL of PV2 solution and incubated at 4° for 5 minutes. Lysates were centrifuged at 13,000 x g for 1 minute, and supernatants transferred to a clean 2.2 ml tube with 600 ul of PV3 and PV4 solutions and vortexed, after which 625 ul of supernatant was repeatedly loaded onto a spin filter and centrifuged at 13,000 x g for 1 minute until all supernatant was loaded onto the filter. 600 ul each of solutions PV5 and PV6 were added, with 1 minute of centrifugation after each, discarding flow-through; tubes were then centrifuged for 2 minutes before each spin filter basket was placed into a clean tube and DNA eluted in 100 ul of RNAse-free water.

### 16S rRNA gene sequencing

Bacterial 16S rRNA gene amplification and library construction were performed according to the 16S Metagenomic Sequencing Library Preparation guide from Illumina (Forest City, CA) with minor modifications. All beads, tubes, and non-enzymatic reagents were treated with UV light for 60 minutes prior to use [23]. Briefly, total DNA was PCR-amplified using primers targeting the 16S V3 and V4 region (Illumina) under the following conditions: 95° C for 5 minutes, followed by 35 cycles of 95° C for 30 seconds, 56° C for 30 seconds, 72° C for 30 seconds, and a final extension of 72° C for 10 minutes [24]. The resulting 16S rDNA amplicons were run on a 1% agarose gel, size selected at 450–500 bp, and gel-purified using QIAquick Gel Purification kit (Qiagen, Valencia, CA). A second round of PCR was performed to add Nextera XT indices (Illumina) to purified amplicons. Indexed PCR products were purified with Ampure XP beads (Beckman Coulter, Inc., Brea, CA), and quantified with Qubit dsDNA system (ThermoFisher Scientific, Waltham, MA). Samples were then normalized and pooled into sequencing libraries at 20 nM then validated on a Bioanalyzer DNA 1000 chip (Agilent, Santa Clara, CA), and sequenced on the Illumina MiSeq with a V3 reagent kit at the Case Western Reserve University Genomics Core Facility.

### Bioinformatics

After the sequencing, the paired-end sequences were processed with QIIME2 package (version 2019.7) [25]. For the subsequent downstream data analysis, we used the Bioconductor phyloseq [26], microbiomeSeq and mixMC [27] packages in R (version 3.6). Initial quality control was checked using fastqc followed by multiqc. The Diverse Amplification Denoise Algorithm (DADA2) pipeline within QIIME2 was used to trim the sequences, dereplicate, filter chimeric sequences and finally merge the paired end reads. DADA2 models corrects for amplification errors, which is more reliable than OTU construction methods. After the quality control visualization, 20bps were trimmed from the beginning of the reads and the reads were truncated to 240bps.

The feature table of amplicon sequence variants (ASV, which is the QIIME2 equivalent of operational taxonomic units [OTUs]), the phylogenetic tree and taxonomy files were constructed within QIIME2. Reads were classified against the SILVA database (silva-132-99-515-806-nb-classifier).

The output of the QIIME2 pipeline was converted to phyloseq object with Qiime2R package (). Phyloseq object was then further filtered and used for creating diversity plots using ggplot2 and with microbiomeSeq R package.

The main statistical approach we used in this study was mixMC, a multivariate analysis framework implemented in mixOmics R package for microbiome data analysis. mixMC helps in identifying OTU features discriminating between multiple groups of samples. mixMC handles compositional and sparse data, repeated-measures experiments and multiclass problems; it highlights important discriminative features, and it provides interpretable graphical outputs to better understand the microbial communities’ contribution to each habitat.

mixMC framework includes unsupervised analyses to visualize diversity patterns with Principal Component Analysis (PCA) and supervised analyses to identify indicator species or determinant microbiota members characterizing differences between habitats or body sites using sparse Partial Least Square Discriminant Analysis, sPLS-DA algorithm.

To apply mixMC we processed data with following steps: a) to the whole data matrix an offset of 1 was added to deal with zeroes after centered log ratio transformation, (b) to remove features with low counts across all samples we prefiltered the raw count data, and (c) we applied a centered log-ratio transformation was to the data.

## Results

### Patient characteristics

A total of 46 patients were enrolled in the study. Of these 46 patients, 32 had cancer of the pancreaticobiliary system including pancreatic carcinoma (n = 25), cholangiocarcinoma (CCA, n = 6), and gallbladder carcinoma (n = 1). Remaining 14 patients were tumor-free volunteers who underwent routine ERCP for benign pancreaticobiliary diseases including acute and chronic pancreatitis, and gallstone disease. Clinical details are shown in Table 1.

**Table 1:** Overall characteristics of all patients.

Patients with malignant pancreaticobiliary diseases			Patients with benign pancreaticobiliary diseases		
	Description	Overall		Description	Overall
n		32	n		14
Patient age, median (range)		66 (37–82)	Patient age, median (range)		56.5 (34–82)
Patient sex, n (%)	Female	11 (34.4)	Patient sex, n (%)	Female	7 (50.0)
	Male	21 (65.6)		Male	7 (50.0)
Patient race, n (%)	Caucasian	28 (87.5)	Patient race, n (%)	Caucasian	13 (92.9)
	African American	3 (9.4)		African American	1 (7.1)
	American Indian/Native Alaskan	1 (3.1)		American Indian/Native Alaskan	0
Tumor type, n (%)	Pancreatic cancer	25 (78.1)	Disease type, n (%)	Acute pancreatitis	3 (21.4)
	Cholangiocarcinoma	6 (18.8)		Biliary stricture	1 (7.1)
	Gallbladder cancer	1 (3.1)		Choledocholithiasis	2 (14.3)
Antineoplastic therapy, n (%)	No	19 (59.4)		Cholelithiasis	1 (7.1)
	Yes	13 (40.6)		Chronic pancreatitis	3 (21.4)
				Jaundice (dilated bile ducts)	2 (14.3)
				Primary sclerosing cholangitis	2 (14.3)
Exposed to antibiotics within the preceding 2 weeks of ERCP, n (%)	Yes	3 (9.4%)	Exposed to antibiotics within the preceding 2 weeks, n (%)	Yes	3 (21.4%)
Deranged Liver Function Tests within the preceding 2 weeks of ERCP, n (%)	Yes	28 (87.5%)	Deranged Liver Function Tests within the preceding 2 weeks, n (%)	Yes	8 (57.1%)

### Microbiome profiling of benign versus malignant pancreaticobiliary disease

**Fig 1:**
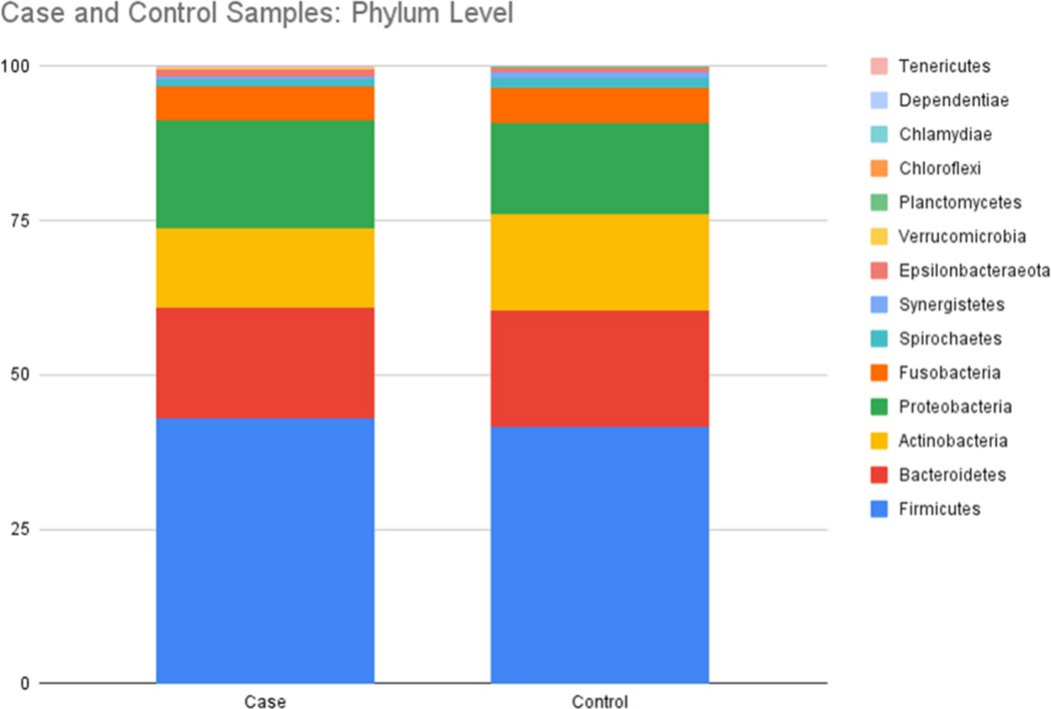
Relative abundance of dominant phyla in bile from patients with benign (control) and malignant (case) pancreaticobiliary diseases.

We explored the microbial signature of bile in patients with benign (controls) and malignant (cases) pancreaticobiliary conditions and compared the relative abundance of microbiota between these two groups. Microbial composition at the phylum level showed five dominant phyla in both cases and controls, namely, Firmicutes, Bacteroidetes, Proteobacteria, Actinobacteria, and Fusobacteria. Relative abundance of these dominant phyla was similar in bile samples from patients with and without cancer of the pancreaticobiliary tract (Fig 1).

**Fig 2:**
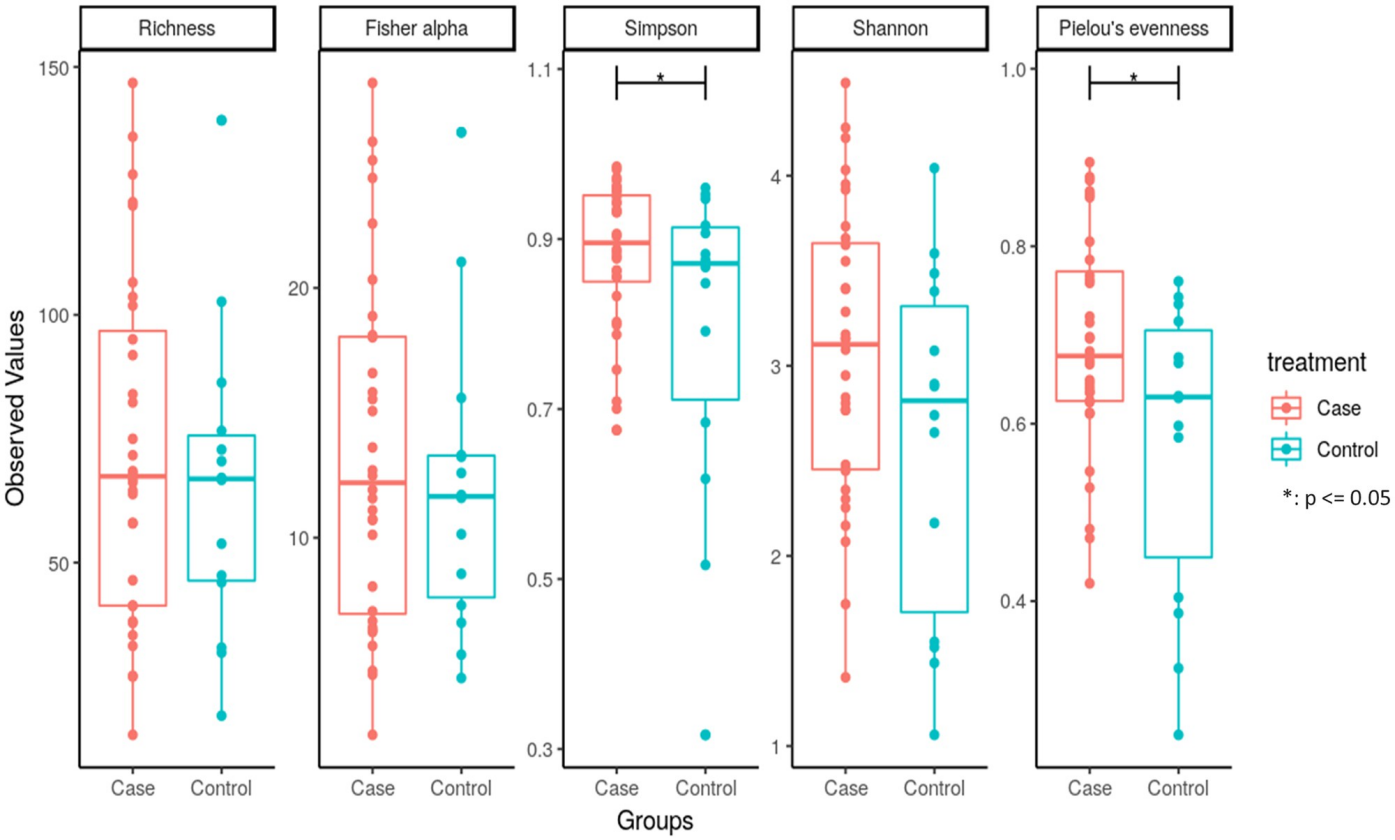
Alpha diversity of biliary microbiota in patients with benign vs malignant pancreaticobiliary diseases.

**Fig 3:**
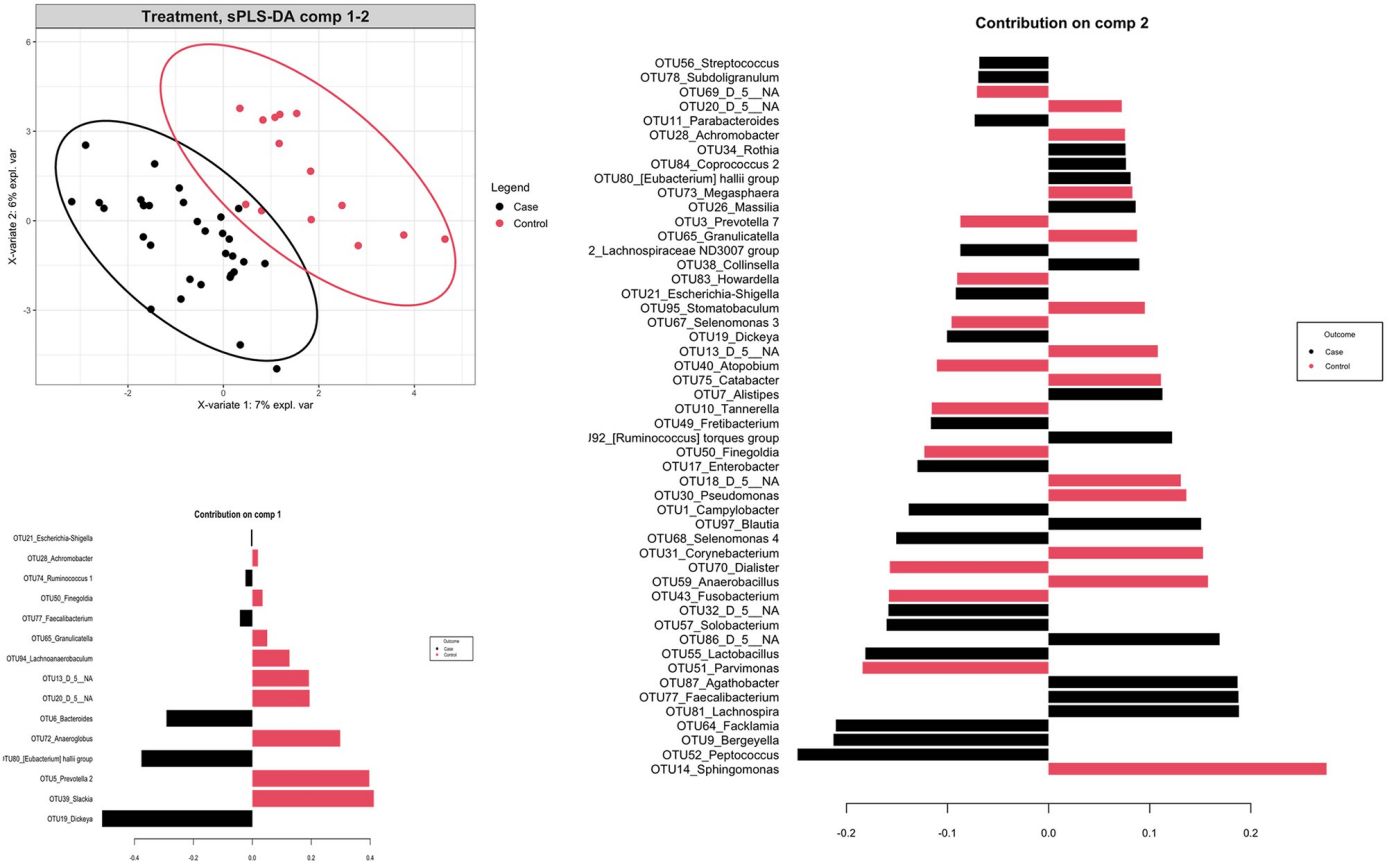
Multivariate analysis and diversity of biliary microbiota in patients with malignant (case) vs benign (control) pancreaticobiliary diseases using mixMC.

**Fig 4:**
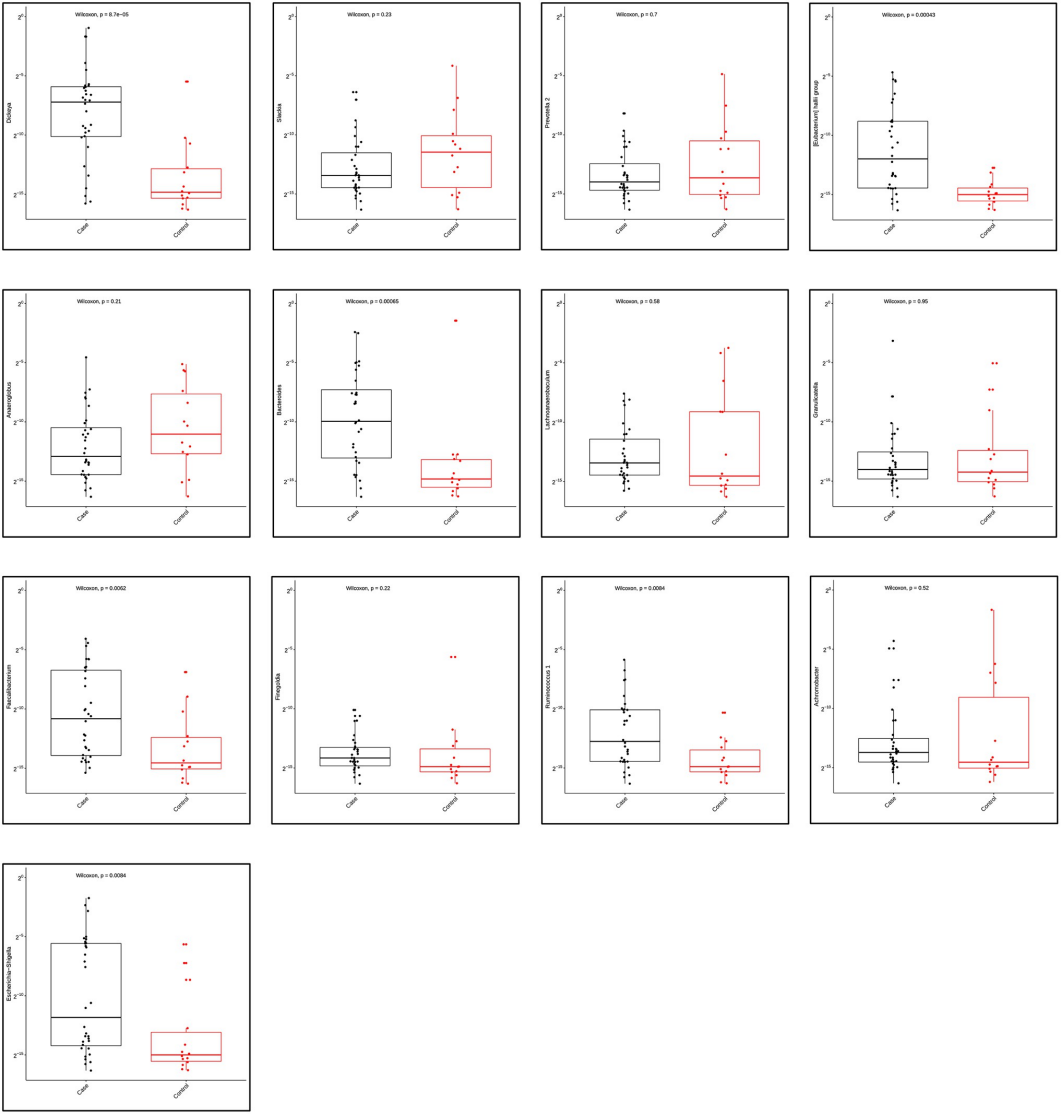
Mann Whitney-U test showing differential abundance of bacterial genera in bile from patients with malignant (case) and benign (control) pancreaticobiliary diseases.

We then compared the richness and diversity of the microbial community in bile samples from both the cases and controls. Overall, there was no significant difference in alpha diversity by Richness, Fisher alpha, and Shannon indices, but significant difference in alpha diversity by Simpson and Pielou’s evenness indices (Fig 2). Furthermore, we used the multivariate approach sparse Partial Least Squares Discriminant Analysis (sPLS-DA) in mixMC to classify OTUs between the cases and controls by agglomerating the data to the Genus level (Figs 3 and 4). mixMC was able to distinctly cluster the data differentiating cases and controls. sPLS-DA showed the effect size of OTUs contributing to the differences between cases and controls (Fig 3). This showed a predominance of the genera *Dickeya* (p = 0.00008), *[Eubacterium] hallii group* (p = 0.0004), *Bacteroides* (p = 0.0006), *Faecalibacterium* (p = 0.006), *Escherichia-Shigella* (p = 0.008), and *Ruminocococcus 1* (p = 0.008) in bile samples from pancreaticobiliary cancers compared to the group of benign pancreaticobiliary diseases (Fig 4).

### Microbiome profiling in pancreatic adenocarcinoma versus cholangiocarcinoma

**Fig 5:**
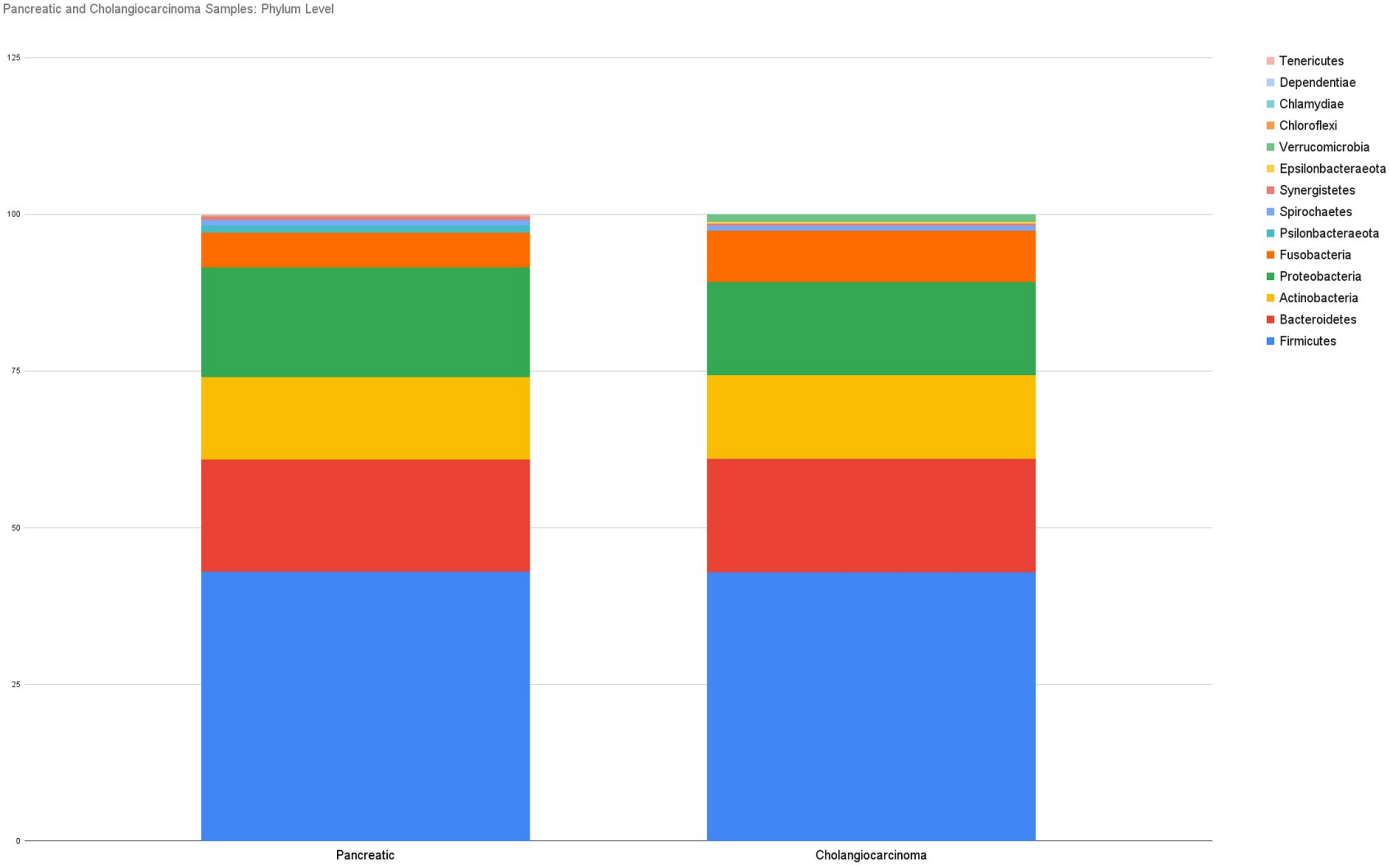
Relative abundance of dominant phyla in bile from patients with pancreatic cancer and cholangiocarcinoma.

We explored the microbial signature of bile in patients with malignant pancreaticobiliary conditions with a focus to compare the relative abundance of microbiota between patients with pancreatic cancer (PC, n = 25) and cholangiocarcinoma (CCA, n = 6). Microbial composition at the phylum level showed five dominant phyla in both subgroups: Firmicutes, Bacteroidetes, Proteobacteria, Actinobacteria, and Fusobacteria. Relative abundance of Firmicutes (43.03% in PC vs 42.86% in CCA), Bacteroidetes (17.85% in PC vs 18.08% in CCA), Proteobacteria (17.5% in PC vs 14.87% in CCA), Actinobacteria (13.11% in PC vs 13.41% in CCA), and Fusobacteria (5.65% in PC vs 8.16% in CCA) was similar in bile samples from patients with pancreatic cancer and those with cholangiocarcinoma (Fig 5).

**Fig 6:**
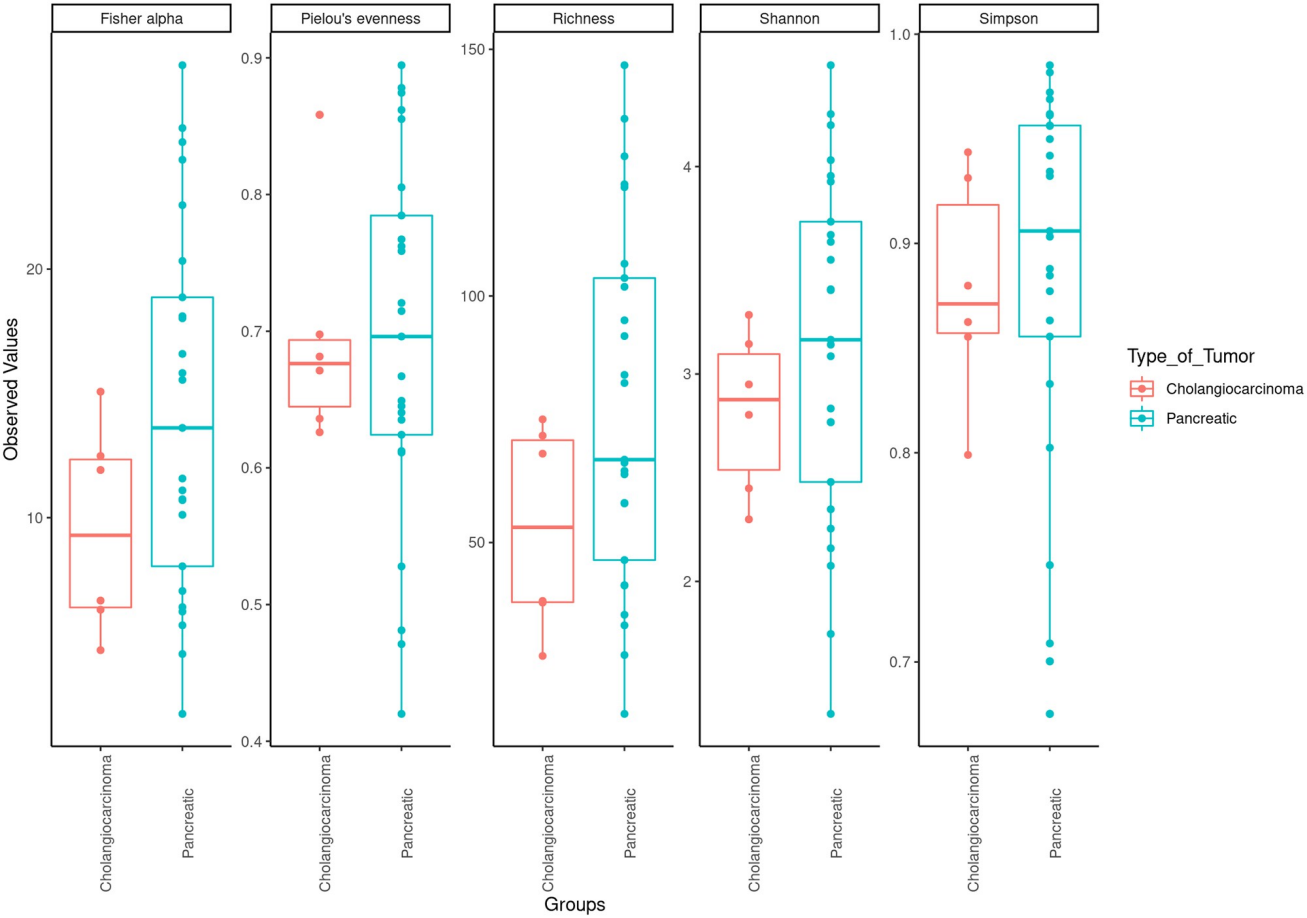
Alpha diversity of biliary microbiota in bile from patients with pancreatic cancer vs cholangiocarcinoma.

**Fig 7:**
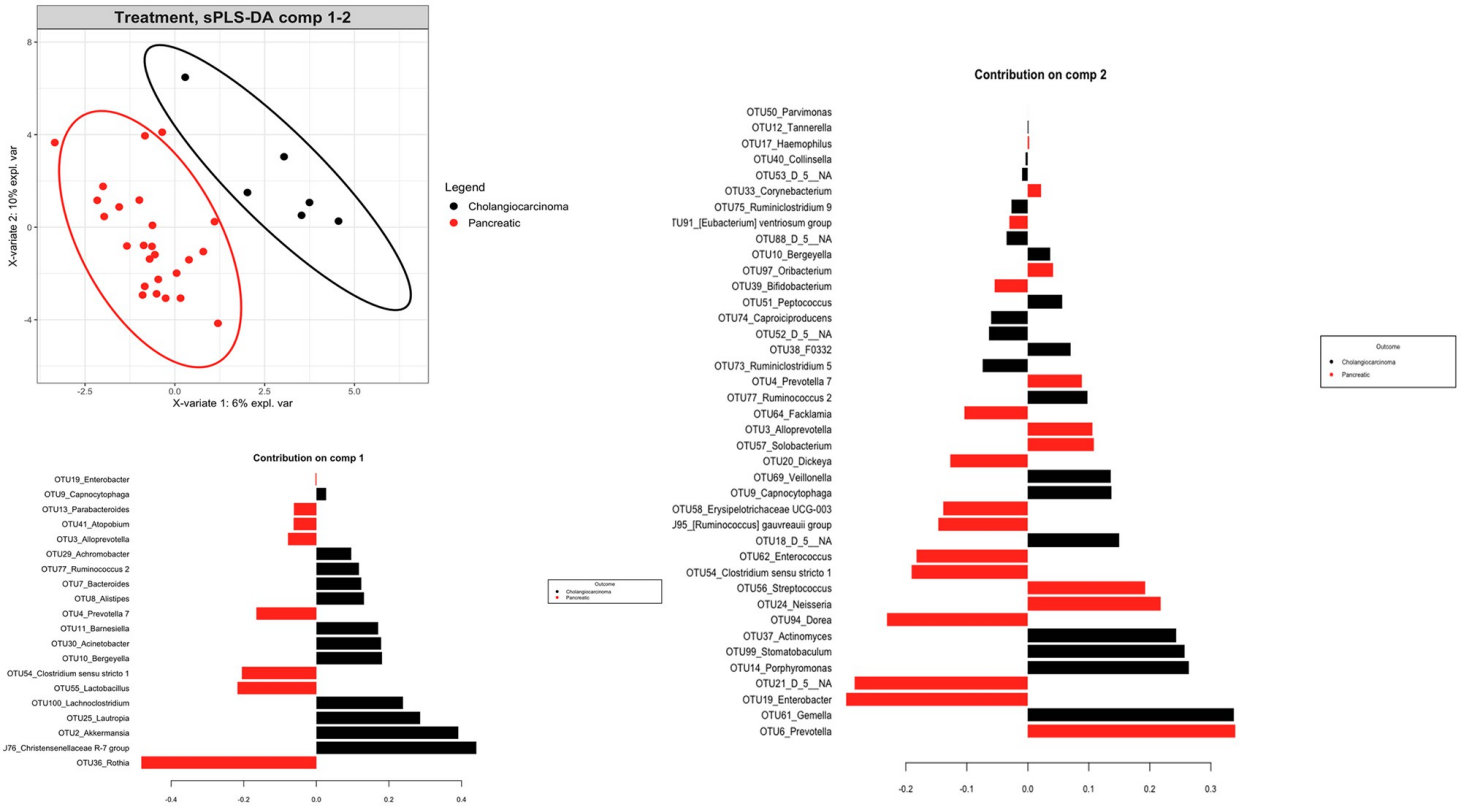
Multivariate analysis and diversity of biliary microbiota in patients with pancreatic cancer versus cholangiocarcinoma using mixMC.

**Fig 8:**
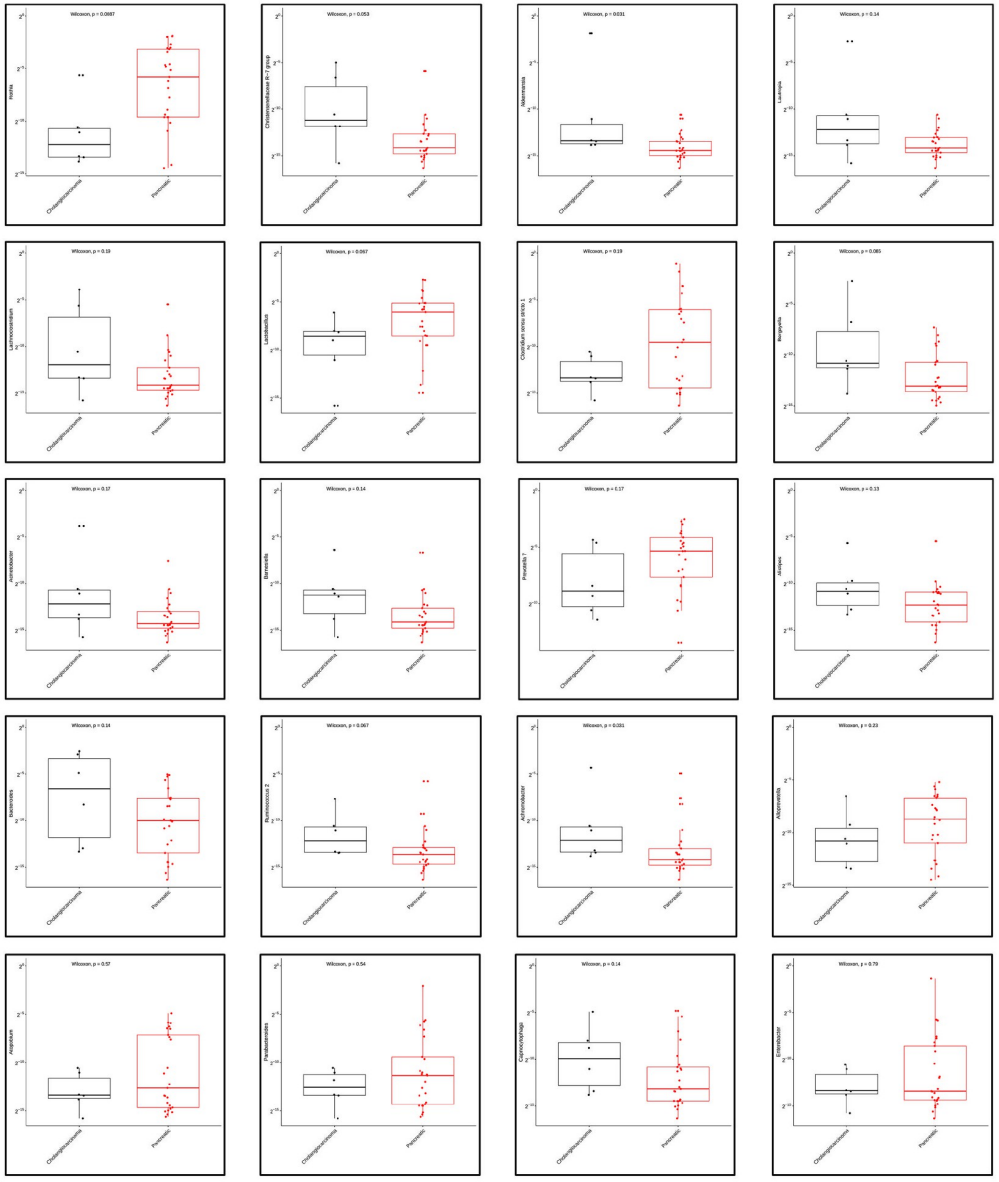
Mann Whitney-U test showing differential abundance of bacterial genera in bile from patients with pancreatic cancer vs cholangiocarcinoma.

We compared the richness and diversity of the microbial community in bile samples from patients with pancreatic cancer, cholangiocarcinoma, and gallbladder cancer by examining alpha diversities. Overall, there was no significant difference in alpha diversity by Richness, Fisher alpha, Simpson, Shannon, and Pielou’s evenness indices (Fig 6). Furthermore, we used the multivariate approach sparse Partial Least Squares Discriminant Analysis (sPLS-DA) in mixMC to classify OTUs in bile samples between patients with pancreatic cancer and with cholangiocarcinoma by agglomerating the data to the Genus level. mixMC was able to identify the differentially abundant microbes between these two subgroups and sPLS-DA clearly separated the two subgroups (Fig 7). Using this analysis, bile samples from patients with pancreatic cancer compared to those with cholangiocarcinoma showed a predominance of genus *Rothia* (p = 0.008). Similarly, bile samples from patiens with cholangiocarcinoma showed a predominance of genera *Akkermansia* (p = 0.031) *and Achromobacter* (p = 0.031) (Fig 8).

## Discussion

Over the past decades, with the increase in next generation sequencing and bioinformatics-based studies investigating the relationship between microbiota and human diseases, we have arrived at an intersection of microbial mechanisms and previously established host-centric cancer hallmarks in our understanding of human cancer [28]. Indeed, an array of metabolites, genotoxins, and antigens derived from microbes influence host immunity, inflammation, energetics, cellular signaling, and metastasis [28]. The ease of obtaining non-invasive biological samples has expanded our understanding of fecal microbiome and its relation to cancer and response to antineoplastic therapies- particularly in gastrointestinal, skin, and lung cancers. Recently, organ-specific commensal microbiomes were revealed in the surveys of samples from non-mucosal sites like pancreas, breast, ovary, lung and skin [14]. Also, evolving literature in microbiomics includes recent evaluations of microbiomes in other liquid niches like bile- both in the benign and malignant cohorts. This has also added to the fundamental knowledge on the composition and activity of the biliary microbiome and its relationship with bile-related disorders [21]. Our pilot study suggests that distinct microbiome signatures in bile are associated with benign and malignant pancreaticobiliary diseases. By using a targeted amplicon sequencing strategy for the 16S rRNA gene, this study demonstrates an underlying dysbiosis of the biliary microbiota among patients with different pancreaticobiliary diseases. Specifically, we found a difference in the relative abundance of OTUs in bile samples between patients with benign pancreaticobiliary diseases and pancreaticobiliary cancers, and between pancreatic cancers and cholangiocarcinomas.

The findings from this study fill an important knowledge gap and are timely for several reasons. First, this study confirms the growing body of evidence that high microbial diversity is present within the biliary milieu of patients with benign and malignant pancreaticobiliary conditions [29–31]. Second, this study shows a high dominance of phyla Firmicutes, Bacteroidetes, Proteobacteria, Actinobacteria and Fusobacteria in the biliary microenvironment and which are also parts of the intestinal microbiota. This is consistent with a recent analysis by del Castillo et. al. that showed the occurrence of these five major phyla in pancreatic tissue samples collected from both the cancer and non-cancer cohorts [13]. This finding correlates with a possibility that biliary microbiota could have their origin from the duodenum despite the sphincter of Oddi. Third, despite the lack of uniform difference in the alpha diversity of the microbial community in bile samples between patients with benign pancreaticobiliary diseases and pancreaticobiliary cancers, a multivariate analysis using mixMC showed a clear separation between these two subgroups. This leads to two hypotheses: that these OTUs may have a role in carcinogenesis, and/or that changes in the microenvironment of benign pancreaticobiliary diseases might differ from the changes in the microenvironment of pancreaticobiliary cancers leading to distinct separation of the OTU clusters. Also, it is not quite clear whether these microbes are cancer-causing oncomicrobes, complicit microbes or so-called innocent bystanders. This observed differential abundance in microbiome raises a possibility that the difference could be due to the nature of the tumor itself by its influence on the gut-liver axis and further alteration of the bidirectional communication between the gastrointestinal tract and the liver via the biliary tract, portal vein and systemic circulation subsequently exposing biliary tract to the gastrointestinal microbiome [32]. Furthermore, this influence on the gut-liver axis might be different for pancreatic cancer which may cause local stasis of the bile through extramural compression of the common bile duct versus cholangiocarcinoma which may cause the stasis in bile flow through intramural obstruction of the common bile duct. A recent study based on mouse models demonstrated that the microbiome is more abundant in the malignant pancreas, as compared with normal controls, and may lead to immune tolerance and lack of response to immunotherapy [33]. In our study, at the genus level, *Dickeya*, *[Eubacterium] hallii group*, *Bacteroides*, *Faecalibacterium*, *Escherichia-Shigella*, *and Rumicococcus1* were significantly predominant in bile samples from patients with pancreaticobiliary cancers as compared to their benign counterparts. Fourth, in this study, multivariate analysis using mixMC clearly separated the microbial taxa at the genus level between patients with pancreatic cancer (increased abundance of *Rothia*) and those with cholangiocarcinoma (increased abundance of *Akkermansia* and *Achromobacter*). This is important because it adds to the growing literature on the microbiomic profile of bile with a focus on cancer. One recent study comparing bile specimens from 10 cholangiocarcinoma patients with 10 patients without malignancy, showed an abundance of *H*. *pylori* in cholangiocarcinoma samples [29]. In our study, *Helicobacter* species were not found to be abundant in cholangiocarcinomasamples. In another study, biliary duct tissue microbiome from liver fluke (*Opisthorchis viverrine*) associated cholangiocarcinoma patients showed significant increase in *Stenotrophomonas* species compared to non-cancer controls, however, this study looked into the microbiome of biliary duct tissue and not the bile *per se* [34].

Not only do our observations here add to the growing literature on microbiome associated with pancreaticobiliary neoplasia, but also importantly, this is one of the first clinical studies to evaluate bile microbiome in pancreaticobiliary diseases, with a focus on cancer. Prior studies have mostly evaluated salivary and gut microbiota in relationship to pancreaticobiliary cancers [10]. In one recent study that investigated bile microbiome in patients with pancreatic cancer, bacteria were detected in only 4 out of 7 bile samples, specifically *Enterobacter* and *Enterococcus* species [35]. *Neisseria* and *Porphyromonas* in oral microbiome have been associated with pancreatic cancer [36, 37] whereas in biliary cancer, there have been multiple reports of *Helicobacter* species with somewhat disparate findings [30]. The role of microbiome in response to immunotherapy for cancer is also being elucidated [7, 8, 38]. *Faecalibacterium*, *Collinsella* and *Enterococcus* species have been reported to be associated with improved response to immunotherapy when present in fecal microbiomes [7, 38]. In our study, *Fecalibacterium* was significantly present in pancreaticobiliary cancer patients when compared to patients with benign pancreaticobiliary diseases.

A strength of this study lies in the collection of specimens solely for the purpose of microbiome analysis and analyzing them to add to the growing body of literature on microbial profile of bile, with a focus on cancer. Moreover, this is the first study to investigate the differential presence and abundance of microbiome in bile in benign compared to malignant pancreaticobiliary diseases, and between pancreatic and biliary cancers.

Our study has several limitations. First, because of the non-randomized nature of the study, our study provides room for the traditional confounders of selection bias. These include but are not limited to the differences in host factors like dietary habits, alcohol consumption, tumor markers and tumor stage, presence of viral hepatitis and liver cirrhosis. We acknowledge the influence that prebiotics, probiotics and antibiotics might have in the biliary microbiome, but although we do not have information on the use of pre- and pro-biotics, only 3 patients each in the benign and malignant pancreaticobiliary disease groups were exposed to antibiotics within the preceding two weeks of bile collection. Second, given the observational design of the study with one-time bile draw and no plan for subsequent bile draws, we do not clearly understand the longitudinal effects of systemic antineoplastic therapies in the biliary microbiome. In a culture-dependent study from patients who had undergone pancreatoduodenectomy for pancreatic cancer, operative bile cultures showed an alteration of biliary microbiome in patients who had received neoadjuvant therapy with an increased likelihood of harboring enterococci and gram-negative bacteria [39]. We do not clearly understand the effect of antineoplastic therapy on the biliary microbiome from our study. Third, the relatively small sample sizes in both benign and malignant pancreaticobiliary subgroups precludes any firm conclusion in this study but do provide good preliminary foundational data. Fourth, this study suggests the presence of a biliary dysbiosis in patients with benign versus malignant pancreaticobiliary diseases and within pancreaticobiliary cancers but does not look into the possibility of dysbiosis in the bile of healthy people without any pancreaticobiliary disorders. This is due to the obvious ethical difficulty of obtaining bile through an invasive procedure like ERCP in healthy volunteers who do not have any pancreaticobiliary disorder, but it would still have added to our understanding on the evolution of pancreaticobiliary cancers if we could have profiled the biliary microbiome among healthy, those with benign diseases and those with cancers. Fifth, despite efforts to reduce contamination during collection of bile, the intervention itself could have introduced bacteria from patients’ oral, gastric, and duodenal flora in the collected sample. And finally, given the cross-sectional nature of the study, this study only profiles the bacterial taxa in bile from cancer patients and non-cancer volunteers and does not establish causality by identifying carcinogenic and non-carcinogenic bacteria.

## Conclusions

This clinical study demonstrates that microbiome analyses of bile may differentiate malignant from benign samples in pancreaticobiliary diseases. Furthermore, distinct microbiome signatures may be associated with pancreatic versus biliary cancers. Taken together, this study adds novel insights to the growing literature on the microbial composition of the biliary microbiota and could help provide a pilot bases for future large-scale studies by utilizing the bile microbiome and its role in human metabolism and health in the diagnosis and treatment of pancreaticobiliary diseases, with a focus on cancer.
